# The Use of Wearables in Clinical Trials During Cancer Treatment: Systematic Review

**DOI:** 10.2196/22006

**Published:** 2020-11-11

**Authors:** Ulrikke Lyng Beauchamp, Helle Pappot, Cecilie Holländer-Mieritz

**Affiliations:** 1 Department of Oncology Rigshospitalet University of Copenhagen Copenhagen Denmark; 2 Faculty of Health University of Copenhagen Copenhagen Denmark

**Keywords:** cancer treatment, wearables, adherence, sensor technology

## Abstract

**Background:**

Interest in the use of wearables in medical care is increasing. Wearables can be used to monitor different variables, such as vital signs and physical activity. A crucial point for using wearables in oncology is if patients already under the burden of severe disease and oncological treatment can accept and adhere to the device. At present, there are no specific recommendations for the use of wearables in oncology, and little research has examined the purpose of using wearables in oncology.

**Objective:**

The purpose of this review is to explore the use of wearables in clinical trials during cancer treatment, with a special focus on adherence.

**Methods:**

PubMed and EMBASE databases were searched prior and up to October 3, 2019, with no limitation in the date of publication. The search strategy was aimed at studies using wearables for monitoring adult patients with cancer during active antineoplastic treatment. Studies were screened independently by 2 reviewers by title and abstract, selected for inclusion and exclusion, and the full-text was assessed for eligibility. Data on study design, type of wearable used, primary outcome, adherence, and device outcome were extracted. Results were presented descriptively.

**Results:**

Our systematic search identified 1269 studies, of which 25 studies met our inclusion criteria. The types of cancer represented in the studies were breast (7/25), gastrointestinal (4/25), lung (4/25), and gynecologic (1/25); 9 studies had multiple types of cancer. Oncologic treatment was primarily chemotherapy (17/25). The study-type distribution was pilot/feasibility study (12/25), observational study (10/25), and randomized controlled trial (3/25). The median sample size was 40 patients (range 7-180). All studies used a wearable with an accelerometer. Adherence varied across studies, from 60%-100% for patients wearing the wearable/evaluable sensor data and 45%-94% for evaluable days, but was differently measured and reported. Of the 25 studies, the most frequent duration for planned monitoring with a wearable was 8-30 days (13/25). Topics for wearable outcomes were physical activity (19/25), circadian rhythm (8/25), sleep (6/25), and skin temperature (1/25). Patient-reported outcomes (PRO) were used in 17 studies; of the 17 PRO studies, only 9 studies reported correlations between the wearable outcome and the PRO.

**Conclusions:**

We found that definitions of outcome measures and adherence varied across studies, and limited consensus among studies existed on which variables to monitor during treatment. Less heterogeneity, better consensus in terms of the use of wearables, and established standards for the definitions of wearable outcomes and adherence would improve comparisons of outcomes from studies using wearables. Adherence, and the definition of such, seems crucial to conclude on data from wearable studies in oncology. Additionally, research using advanced wearable devices and active use of the data are encouraged to further explore the potential of wearables in oncology during treatment. Particularly, randomized clinical studies are warranted to create consensus on when and how to implement in oncological practice.

## Introduction

Technology expansion over the past decade, along with the use of various sensors and electronic devices and the arrival of more advanced devices, has led to new possibilities [1-3]. The potential use of sensor technology in the health care setting is wide and covers aspects suitable for many purposes [4,5]. In this review, we focus on the use of wearables in clinical trials during cancer treatment.

A wearable is a device with a sensor that can collect health-related data remotely [6]. Depending on the device design, it can be worn in different ways, either on the wrist, upper arm, around the waist, fastened to the hip, or on another location of the body. Wearables can collect information on various biometric data points (eg, heart rate, respiratory rate, blood oxygen saturation, sleep pattern, or body temperature) [7]. This information can be used alone or in combination with other information [eg, patient-reported outcome (PRO) or other patient-generated health data] to evaluate or estimate a clinical outcome; hospitalization, adverse events, performance status, and physical activity can be used as a clinical outcome. The information can be used for home monitoring with feedback to the clinician or for self-management by the patient. The collection of data from the wearable can be either offline or in real time, depending on the type of feedback wanted for the user (eg, the patient or clinician).

In oncology, wearables may offer new vital information about patients, which can potentially lead to better management of cancer treatment [7]. This is important both in the aspect of precision care and economy, with cancer being the second leading cause of death globally [8]. Wearables make it possible to monitor patients at home in their own environment, compared to monitoring only at in-clinic visits [9]. Tracking data in the patient’s own environment might allow the patient to continue their normal routines while their health data is being transmitted to a database or directly to the clinician. The possibilities are appealing in oncology from a “work smarter” approach and, more importantly, for the general improvements in the quality of life they may offer to patients with cancer of [10,11]. At present, there is little clinical evidence showing how wearables might improve the cancer pathway for patients with cancer during treatment [12].

To understand the potential use of wearables, it is relevant to capture what the wearable’s objective outcome is, what effect or role it has on the clinical outcome, and what it can be used for in a medical setting [7]. Evaluating the patient’s adherence to the wearable and defining valid data helps to ensure that new technologies are introduced into clinical practice with a focus on the patient’s perspective. Limited consensus and guidelines exist for designing or reporting trials using wearables as part of the intervention—but this research area is getting increased attention [13,14]. One initiative is the Clinical Transformation Initiative (CTTI), which has issued recommendations regarding the appropriate use of mobile technology in clinical trials [13]. They have also initiated a database on feasibility studies in clinical trials [15]. The database is not limited to one specific disease group but only includes feasibility studies [15].

The purpose of this review is to explore the use of wearables in clinical trials during cancer treatment, with a focus on adherence and the setting. 

## Methods

### Study Design and Search Strategy

Systematic searches were performed in PubMed and EMBASE. Both databases were searched prior and up to October 3, 2019, with no limitation in the date of publication. Searches consisted of cancer/neoplasm keywords and terms for wearable devices.

In PubMed, the search consisted of the medical-subject-heading (MeSH) terms “neoplasms,” “medical oncology,” “surgical oncology,” and “wearable electronic devices,” along with a combination of free-text words for the topics “oncology,” “cancer,” “wearable device,” “accelerometer,” and “actigraph.” In EMBASE, the search included categorized terms for neoplasms and electronic monitoring devices such as “neoplasm,” “patient monitoring,” and “electronic device.” Additional search terms “ambulatory monitoring” and “telemedicine” were added. The search was limited to articles published in English. The search strategy is shown in Multimedia Appendix 1. The review was registered at PROSPERO (International prospective register of systematic reviews) ID number CRD42020154386 before data extraction was initiated [16].

### Criteria for Inclusion of Studies

Studies found with the selected search strategy were screened by title and abstract, which was performed independently by 2 reviewers who were blinded to each other’s decisions. Cases of disagreement about whether to include or exclude a study were decided through a consensus decision. The included studies had their full text assessed for eligibility; disagreements were resolved by consensus, which was achieved in all cases.

For a study to be included, it had to be written in English and be either a randomized controlled trial (RCT), observational study, or pilot study/ feasibility study. Patients had to be 18 years of age or older and diagnosed with a solid malignant tumor. It was mandatory that studies took place during active cancer treatment; treatment could be either radiation or antineoplastic treatment, such as chemotherapy or targeted therapy. Studies investigating all types of wearables were considered eligible if they had an objective measure. Studies had to include a description of adherence to the wearable to be eligible for inclusion.

Exclusion criteria were studies registered as protocol descriptions, study protocols, abstracts from conferences, editorials, letters, or case reports. Also excluded were studies in which patients were cancer survivors or had hematologic malignancies and were treated with endocrine therapy only or surgery only. If the wearable devices were worn only pretreatment, the study used hearing aids as the wearable device, or wearables were used as treatment or for diagnostics, then these studies were also excluded.

**Figure 1:**
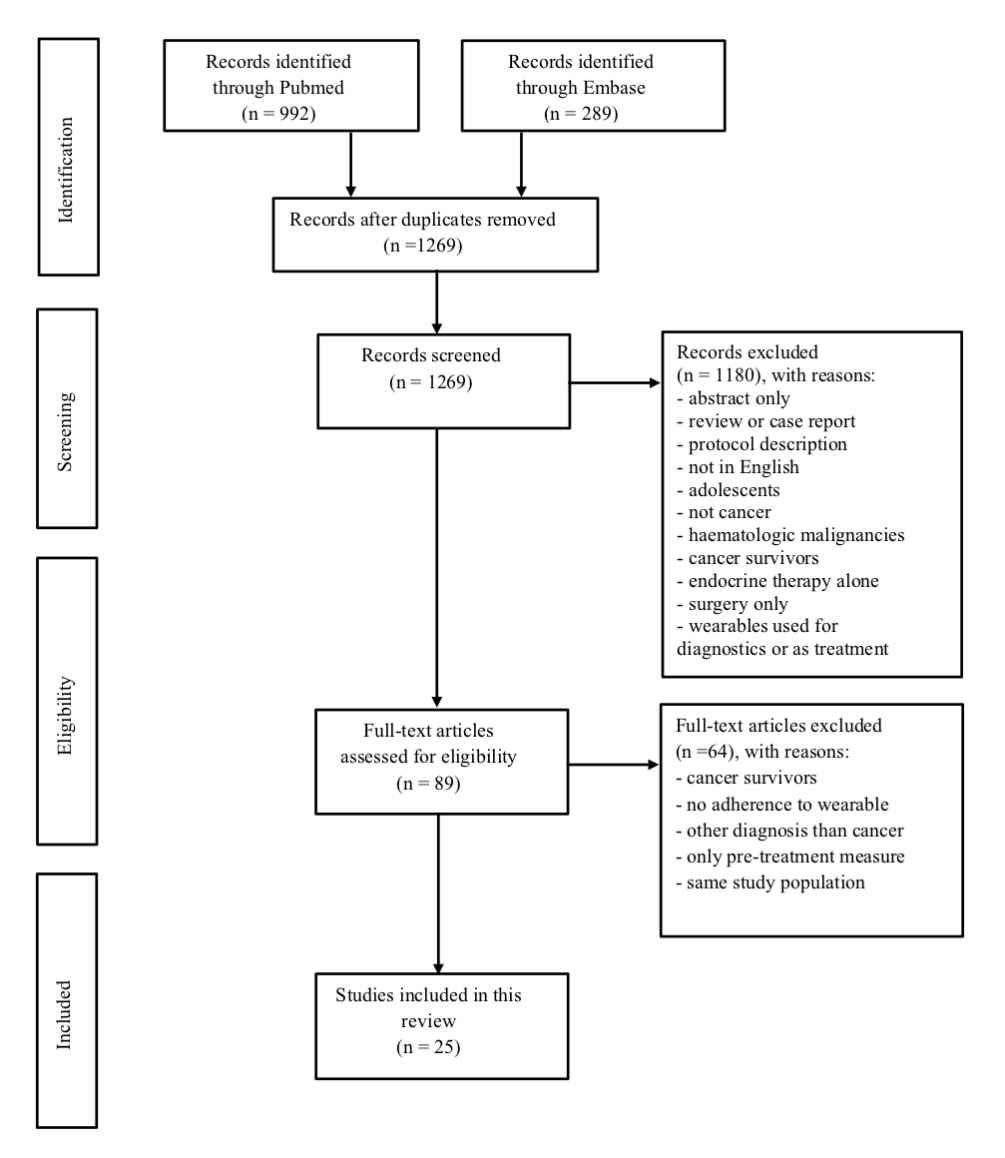
PRISMA flow diagram of the screening and selection of studies.

The search is graphically presented according to the PRISMA flow diagram (Figure 1).

### Data Extraction

The following study characteristics data were extracted: study title, author, year of publication, country, study design, number of patients included, and main objectives. Study population information that was extracted included age group, cancer type, treatment type, and intent of treatment. Study data regarding the adherence to the wearable were the type of wearable used (hardware, software), placement, device outcome, planned wear time, valid wear time, and adherence to the wearable. For the purposes of this review, the device outcome was defined as the objective measures used in the study (eg, step count); planned wear time was defined as the time period that patients were supposed to use the device; and valid wear time was defined as the minimum wear time for data inclusion, extracted if available (eg, ≥10 hours/day). Adherence could either be the percentage of patients wearing the wearable for the period, the percentages of patients with evaluable sensor data, or the percentages of total evaluable days. Study outcomes were extracted and thematically grouped into wearable outcome, PRO, and clinical outcome. Wearable outcome was defined as circadian rhythm, physical activity (PA), skin temperature, and sleep. PRO topics were quality of life, PA, mental health, symptom registration, and others. Clinical outcomes included adverse events, performance status (PS), and hospitalization.

When reading through the full text of included studies, synonyms for “wearable” were registered and extracted from each study.

### Outcomes and Analysis

The review was conducted to give a descriptive presentation of the use of wearables in clinical trials. The primary outcome was adherence to the wearable. The secondary outcomes were the study outcomes: the wearable outcome, the PRO, and the clinical outcome. The wearable outcome was subtracted from the device outcome by the authors, and thematically grouped as a wearable outcome. We also investigated whether the studies reported a relationship between the wearable outcome and the PRO. All data were presented descriptively.

### Ethical Considerations

This review did not require national or institutional approval.

## Results

The search strategies were performed in PubMed and EMBASE on October 3, 2019. A total of 1281 studies were identified through the searches. There were 12 duplicated records, which were excluded, leaving 1269 studies eligible for screening. Titles and abstracts were examined, which resulted in the exclusion of 1180 studies that did not meet our inclusion criteria. The remaining 89 studies were evaluated for eligibility. Through full-text access, 64 other irrelevant studies were excluded, leaving a total of 25 studies to be reviewed for the purpose of this review. This process of study screening and selection is illustrated in a PRISMA flow chart (Figure 1).

Study characteristics are presented in Table 1, which shows a wide heterogeneity with respect to primary cancer sites (range, 1-8 sites), treatment-type specifics (eg, whole-brain radiotherapy, to all treatments allowed), sample sizes (n=7-180), and age groups (22-94 years). In terms of cancer diagnosis, 13 of the 25 studies included patients with breast cancer, 7 having breast cancer exclusively. Gastrointestinal (GI) cancer was featured in 12 of the 25 studies, 4 of which studied patients with GI exclusively. Lung cancer was present among 10 of the 25 studies, 4 featuring this diagnosis exclusively. For 19 of the 25 studies, the patients were treated with curative intent. For most (17/25) of the included studies, treatment was chemotherapy. Of the 25 studies included, a total of 12 were pilot/feasibility studies, 10 were observational studies, and 3 were randomized controlled trials (Table 1).

In 5 of the 25 studies, the planned wear time was ≤7 days, 13 were between 8-30 days, 5 were between 31-90 days, and in 2 studies, the planned wear time was over 90 days (Table 2).

**Table 1:** Characteristics of included studies, n=25.

Study (year)	Country	Primary cancer site	Treatment type	Sample size, n	Age group (range or mean [SD])	Study type
Broderick et al (2019) [17]	United States	Mixed	Chemotherapy	42	24-72	Pilot / feasibility study
Champ et al (2018) [18]	United States	Breast	Radiotherapy	10	52-79	Pilot / feasibility study
Chevalier et al (2003) [19]	France	Gastrointestinal	Chemotherapy	10	43-73	Pilot / feasibility study
Dean et al (2013) [20]	United States	Lung	Chemotherapy	35	48-94	Observational study
Dreher et al (2019) [21]	United States	Breast	Chemotherapy	65	29-72	Observational study
Edbrooke et al (2019) [22]	Australia	Lung	Mixed	92	63 (12.3)	Randomized controlled trial
Gupta et al (2018) [23]	United States	Mixed	Systemic therapy	24	54 (12.5)	Pilot / feasibility study
Innominato et al (2016) [24]	United Kingdom	Mixed	Chemotherapy	31	35-91	Pilot / feasibility study
Li et al (2019) [25]	China	Breast	Adjuvant chemotherapy	180	22-74	Observational study
Low et al (2017) [11]	United States	Gastrointestinal	Chemotherapy	14	40-74	Pilot / feasibility study
Lowe et al (2014) [26]	Canada	Mixed	Radiotherapy (whole brain)	31	63.5 (10.4)	Observational study
Mouri et al (2018) [27]	Japan	Mixed	Chemotherapy	30	70-84	Pilot / feasibility study
Nyrop et al (2018) [28]	United States	Breast	Chemotherapy	100	24-64	Observational study
Ohri et al (2019) [29]	United States	Lung	Chemo-radiotherapy	50	38-90	Observational study
Ohri et al (2017) [30]	United States	Mixed	Chemo-radiotherapy	38	33-82	Pilot / feasibility study
Ortiz-Tudela et al (2014) [31]	France	Mixed	Chemotherapy	49	35-90	Observational study
Parker et al (2019) [32]	United States	Pancreas	Chemotherapy; chemo-radiotherapy	50	66 (8)	Observational study
Roche et al (2014) [33]	France	Gastrointestinal	Chemotherapy	16	51-89	Pilot / feasibility study
Roscoe et al (2002) [34]	United States	Breast	Chemotherapy +/- radiotherapy	102	34-79	Randomized controlled trial
Sarna et al (2001) [35]	United States	Mixed	Radiotherapy	7	48-74	Pilot / feasibility study
Savard et al (2009) [36]	United States	Breast	Chemotherapy	95	34-79	Observational study
Solk et al (2019) [37]	United States	Breast	Chemotherapy	67	31-71	Observational study
van der Meij et al (2012) [38]	The Netherlands	Lung	Chemo-radiotherapy	40	39-80	Randomized controlled trial
Vassbakk-Brovold et al (2016) [39]	Norway	Mixed	Chemotherapy	66	59 (11)	Pilot / feasibility study
Wright et al (2018) [40]	United States	Gynaecological	Chemotherapy	10	60 (11)	Pilot / feasibility study

**Table 2:** Description of wearables and adherence.

Planned wear time interval and study (year)		Hardware / software	Device outcome	Planned wear time / valid wear time	Adherence description
≤**7 days**					
	Chevalier et al (2003) [19]	Actigraph, Ambulatory Monitoring Inc / Action 3.8	Rest activity cycle (movements/period)	3 days / 3 days	100% (10/10) of the patients wore the device for the full period
	Dean et al (2013) [20]	Motionlogger actigraph / Action 3	Sleep efficiency (%) Sleep (hours) Wake after sleep onset (minutes)	7 days / not reported	86% (30/35) of the patients wore the device for the full period
	Lowe et al (2014) [26]	activPAL^a^ / not reported	Position time (hours/day) Energy expenditure (metabolic equivalent of task [MET] h/day) Step count (steps/day)	7 days / not reported	77% (24/31) of the patients provided evaluable sensor data between 3 and 7 days
	Roscoe et al (2002) [34]	Mini-Motionlogger Actigraph / Action 3	Circadian consistency (I<O^b^) Daytime activity level (minutes) Sleep (%)	72 hours at 2 timepoints / not reported	89% (91/102) provided evaluable sensor data at second cycle of chemotherapy 44% (45/102) provided evaluable sensor data at fourth cycle of chemotherapy
	Vassbakk-Brovold et al 2016) [39]	SenseWear Armband Pro3 or SenseWear Armband Mini^a^ / SenseWear version 6.1 for Pro3 and version 7.0 for Mini	Physical activity (minutes/week) recorded in 1-minute epochs	5 days / ≥19.2 hrs, for ≥1 day	79 % (66/84) of the patients wore the device for the full period
**8-30 days**					
	Edbrooke et al (2019) [22]	SenseWear accelerometer^a^ / not reported	Step count (steps/day) Number of 10+ minutes step bouts/day Duration of 10+ minutes bouts (minutes) Cadence of 10+ minutes bouts (steps/min)	7 days at 3 timepoints / 8hrs/day, for ≥4 days	87% (80/92) of the patients provided evaluable sensor data at baseline 71%(65/92) of the patients provided evaluable sensor data at 9 weeks 60% (55/92) of the patients provided evaluable data at 6 months
	Innominato et al (2016) [24]	Micro Motionlogger / Action 4	Circadian rest-activity (I<O^b^)	30 days / not reported	Evaluable sensor data were available in 75 % of the total days (653/874)
	Li et al (2019) [25]	GENEActiv Original / not reported	Sleep efficiency (%) Sleep duration (minutes) Nighttime total wake time (minutes)	7 days at 3 timepoints / ≥5 days per timepoint	97% (175/180) of the patients provided evaluable sensor data at T2 76% (136/180) of the patients provided evaluable sensor data at T3
	Low et al (2017) [11]	Fitbit Charge HR / not reported	Step count (steps/day) Floors climbed (n) Sleep (minutes) Awakenings (n) Time in bed (minutes)	4 weeks / not reported	Evaluable sensor data were available in 75 % of the total days (295/392 days)
	Mouri et al (2018) [27]	Kenz Lifecorder‐GS^a^ / Lifelyzer‐05 coach	Step count (steps/day) Physical activity (minutes/day) (physical activity was rated ≥1.8 METs)	7 days at 3 timepoints / ≥5 hrs/day	93% (28/30) of the patients wore the device for the full period
	Ohri et al (2019) [29]	Garmin Vivofit ^a^ / not reported	Step count (steps/day)	Up to 3 weeks / not reported	Evaluable sensor data were available in 94 % of the total days (741/791)
	Ortiz-Tudela et al (2014) [31]	Mini-Motionlogger Actigraph / Action 4	Rest-activity (I<O^b^) Wrist accelerations (acc/minute)	10-14 days split into 4 periods of 3-4 days / not reported	86% (42/49) of the patients provided evaluable sensor data the full period
	Roche et al (2014) [33]	Mini-Motionlogger and VitalSense / Action 4, version 1.10	Rest-activity (I<O^b^) Wrist accelerations (acc/minute) Skin surface temperature (°C/minute)	12 days split into 3 periods of 4 days/ not reported	100% (16/16) of patients provided evaluable sensor data at baseline 63% (10/16) of patients provided evaluable sensor data during therapy and after therapy administration
	Sarna et al (2001) [35]	Actiwatch 2 / not reported	Wrist movement (n/second) Physical activity (15-minute intervals)	5 days at 2 timepoints/ ≥3 days per timepoint	100% (7/7) of the patients wore the device the full period
	Savard et al (2009) [36]	Actillume / Action 3	Circadian rhythm variables (calculated from orientation and movement)	72 hrs at 7 timepoints/ not reported	91% (86/95) of patients provided evaluable sensor data at baseline (first cycle of chemotherapy week 1: 80%, week 2: 73% and week 3: 76%; fourth cycle of chemotherapy week 1: 74%, week 2 63% and week 3: 68%)
	Solk et al (2019) [37]	ActiGraph, model wGT3X-BT / ActiLife, version 6.13.3	Activity data (1-minutes intervals)	10 days at 3 timepoints / ≥10 hrs/day	84% (63/75) of the patients provided evaluable sensor data for the full period
	van der Meij et al (2012) [38]	PAM accelerometer, model AM101^a^ / not reported	Physical activity (index score, 3 points reflects about 10 min of walking)	7 days at 3 timepoints / ≥3 full days	65% (26/40) of the patients wore the device for the full period
	Wright et al (2018) [40]	Fitbit Zip and Fitbit Charge 2 / Fitabase	Step count (steps/day) Heart rate	30 days / ≥4 days/week	90% (9/10) of the patients wore the devices for the full period
**31-90 days**					
	Broderick et al (2019) [17]	Microsoft Band 2 / not reported	Step count (steps/day) Heart rate Calories (calories/hour)	60 days / ≥6 hrs/day	Evaluable sensor data were available 86 % of the days (only day 1-14 included)
	Champ et al (2018) [18]	Misfit Shine^a^ / not reported	Step count (steps/day) Calories (calories/day) Walking distance (miles) Sleep (hours)	10 weeks / not reported	90% (9/10) of the patients wore the device for the full period
	Gupta A et al (2018) [23]	Fitbit Flex / not reported	Step count (steps/day) Physical activity (sedentary minutes/day) Sleep (minutes)	12 weeks / ≥1 steps/day recorded	96% (23/24) wore the device for >50% of the period
	Nyrop et al (2018) [28]	Fitbit Zip^a^ / not reported	Step count (steps/day)	6-12 weeks / ≥3 weeks	79% (100/127) of the patients provided evaluable sensor data
	Ohri et al (2017) [30]	Garmin / not reported	Step count (steps/day)	Up to 80 days / 80% of the days	Evaluable sensor data were available 94 % of the days
**>91 days**					
	Dreher et al (2019) [21]	Fitbit Charge HR or Fitbit Charge 2 / Fitabase	Step count (steps/day) Heart rate Sleep data	Up to 270 days / ≥10 hrs/day	Evaluable sensor data were available in 45% of the days across 9 months
	Parker et al (2019) [32]	ActiGraph GT3X+^a^ / ActiLife Software, Version 6	Physical activity (minutes/week) (1-min epochs)	14 days at each therapy phase / ≥10 hrs/day, for ≥7 days per timepoint	88 % (44/50) of the patients provided evaluable sensor data

^a^Placement other than wrist (anterior mid-thigh, hip, triceps muscle waist, not reported).

^b^bI<O is computed as the percentage of activity epochs when in-bed (I), whose values are lower than the median level of activity when out-of-bed(O).

Adherence data, presenting how many patients were able to use or collect data from the wearable device, or how many evaluable days the wearable was worn, were collected. Adherence varied across studies, from 60%-100% and 45%-94%, respectively, but was differently measured and reported. Valid wear time was defined in 16 of the 25 studies. Different hardware and software were used. The most frequent placement of the wearable was the wrist.

In Table 3, study outcomes were grouped as wearable outcome, PRO, and clinical outcome, and their respective subtopics can be seen for each included study, showing that 1 study could have more than 1 topic assigned. Of the 25 studies included, 19 had the purpose of monitoring PA (wearable outcome) as an outcome. The second most frequent topic among wearable outcomes was circadian rhythm, monitored in 8 studies. With respect to PRO, 9 studies examined quality of life, 7 studied mental health, 7 studied physical activity, and 8 looked at symptoms. Clinical outcomes were few, 4 comprising performance status, 2 looking at adverse events, and 2 studying hospitalization (Table 3).

**Table 3:** Study outcomes

Cancer type and study (year)		Wearable outcomes					Patient-reported outcomes					Clinical outcomes			
			Circ. rhythm^a^	Phys. activity^b^	Skin temp.^c^	Sleep	Mental health	Phys. activity	QoL^d^	Symptoms	Other	Adverse events	Perf. status^e^	Hospitalization	Other
**Breast**															
	Roscoe et al (2002) [34]		✓	✓		✓	✓				✓		✓		
	Savard J et al (2009) [36]		✓			✓									
	Champ et al (2017) [18]			✓		✓									
	Li et al (2019) [25]		✓	✓											✓
	Nyrop et al (2018) [28]			✓			✓	✓	✓	✓	✓	✓	✓		✓
	Dreher et al (2019) [21]		✓	✓											
	Solk et al (2019) [37]			✓			✓	✓		✓	✓				
**Gastrointestinal**															
	Chevalier et al (2003) [19]		✓												
	Roche et al (2014) [33]		✓		✓										
	Low et al (2017) [11]			✓		✓				✓					
	Parker et al (2019) [32]			✓				✓							✓
**Gynecological**															
	Wright et al (2018) [40]			✓					✓	✓					
**Lung**															
	van der Meij et al (2012) [38]			✓					✓						
	Dean et al (2013) [20]					✓	✓		✓		✓				
	Edbrooke et al (2019) [22]			✓			✓	✓	✓	✓					
	Ohri et al (2019) [18]			✓										✓	✓
**Mixed**															
	Sarna et al (2001) [35]			✓			✓			✓	✓				
	Ortiz-Tudela et al (2014) [31]		✓									✓			
	Lowe et al (2014) [26]			✓				✓	✓	✓					
	Innominato et al (2016) [24]		✓							✓					
	Vassbakk-Brovold et al (2016) [39]			✓				✓							✓
	Ohri et al (2017) [30]			✓					✓					✓	✓
	Gupta et al (2018) [23]			✓		✓	✓		✓		✓		✓		
	Mouri et al (2018) [27]			✓					✓						
	Broderick et al (2019) [17]			✓				✓			✓		✓		

^a^Circ. rhythm: Circadian rhythm.

^b^Phys. activity: Physical activity.

^c^Skin temp.: Skin temperature.

^d^QoL: Quality of life.

^e^Perf. status: Performance status.

Of the 17 PRO studies, only 9 studies reported correlations between the wearable outcome and the PRO (Table 4). It was primarily physical activity, which was compared with the PROs (7/9).

Synonyms for “wearable” were also collected for each study (data not shown) while reading through the full text, which reflected both the terms used to address the technology in general and the terms describing the actual device used in the study. The most commonly used term was “accelerometer,” which was used in 12 studies; next was “actigraph,” which was mentioned in 8 studies. The term “tracker” was used in 6 studies as a part of several terms, including “activity tracker,” “wearable activity tracker,” and “fitness tracker.” The latter term was used similarly to “monitor,” which was mentioned in 9 studies.

**Table 4:** Studies that reported relationships between wearable outcomes and patient-reported outcomes (PRO; n=9).

PRO	Wearable outcome			
	Circadian rhythm	Physical activity	Skin temperature	Sleep
Mental health	[34]	[28],[22], [23]	—^a^	[23]
Physical activity	—	[28], [22], [26], [39], [17]	—	—
Quality of life	—	[28], [22], [26], [23]	—	[23]
Symptoms	—	[28], [11], [22], [26]	—	[11]
Others	[34] (fatigue)	[28] (fatigue), [23] (fatigue), [17] (fatigue, sleep)	—	[40] (sleep), [23] (fatigue)

^a^No relationship reported.

## Discussion

### Summary of Findings

The use of wearable sensor devices has become a popular self-awareness gadget for many people today, especially when it comes to measuring physical activity [41]. In this review, we demonstrate the heterogeneous use of wearables during cancer treatment reported in research studies. In a search of the literature, 1269 studies were identified, of which 25 were included in our review. These studies represented different cancer types, with most focusing on mixed cancer types or breast cancer solely. Treatment given in the studies was primarily chemotherapy. Study types were pilot/feasibility, observational, and randomized controlled studies with sample sizes varying from 7 to 180 patients. All studies included in the review used a wearable with an accelerometer, but monitoring duration varied (3-270 days); and even though most studies (19/25) had physical activity as the wearable outcome, the device outcome for physical activity varied. With respect to adherence to using the wearable, a considerable variation was seen. Of the 17 PRO studies, 9 studies made comparisons between wearable outcome and PRO. In general, we noticed a broad variation in study designs, definitions, and outcomes within this field. This was also reflected in the number of synonyms for “wearable” used as terms to address the technology in general and as terms to describe the actual device used in a study.

### Our Focus

Other studies have reviewed the use and effects of eHealth tools, such as those for patient self-reporting of medication management and use, and have concluded that more high-quality research is needed before standard implementation of such tools can occur [42]. In oncology, there is a growing urge to include patients in the management of their own illness, making wearable devices a valuable tool in cancer therapy. However, technical and clinical feasibility are essential aspects to explore [15], as the device measurements depend on patients using them. In this review, we have additionally focused on a somewhat understudied issue—adherence. We report adherence in relation to wear time and report how many patients were able to use or collect data with the wearable device, or how many evaluable days the wearable was worn. Adherence appears to have a wide range, and no data are available on missing wear time, which leaves us questioning if patients only wear wearables when they feel fit or when it is convenient. Thus, before designing large intervention studies using wearables, one must consider defining minimum wear time or conditions with mandatory wear time, since changes in these parameters might influence results [13,14,43].

### Strengths and Limitations

To our knowledge, this is the first review of the use of wearables in clinical trials during cancer treatment. This review was limited to studies that included adherence as an issue, but was not restricted to specific types of wearables. However, we and others believe that the choice of wearable outcome is highly important. Determination of which variables to measure is crucial to ensure the purpose of the wearable when incorporated into patients’ daily routines [1]. Outcomes must be of specific value regarding the individual patient’s course of disease. We agree with other researchers that it is unnecessary to monitor many different kinds of variables if the outcome is not going to have a significant impact on the patient’s treatment [41]. At present, most studies tend to choose PA as the wearable outcome, but this may be because knowledge from the field of fitness and fitness training has grown [44]. Other wearable outcomes might be more relevant in the cancer setting, but this remains to be studied. Further, the objective measure of PA across studies varies. Of 9 studies comparing the wearable outcome and PRO, only 2 were randomized controlled trials. Further investigations in this area is needed before conclusions can be drawn. We suggest that future research include measurements of relevant, well-defined outcomes and be based on guidelines within the field, where such exist [45,46].

Many different terms are being used to describe electronic devices for use in health care [47], which can make it difficult to get an overview of the field and to keep up with how far research has come. In general, the terms used to describe the technology differ, from being very specific (eg, “wearable activity tracker” or “physical activity monitor”) to being broad, and covering several types of devices (eg, “telehealth,” “mobile health,” “wearable devices,” and “remote patient monitoring”) [7,48]. Consensus regarding the terms used could be helpful for both indexing studies and categorizing results by the specific types of devices used; such uniformity would improve research in the field and probably lead to more and improved knowledge.

The patient populations represented in this review mostly reflect breast and mixed cancer populations. Additionally, most studies are from the United States. It is questionable if results from such studies can be transferred to other diagnoses, countries, and cultural settings.

### Implications for Future Research

As in this review, overall adherence to wear time or to wearable device interventions in general are difficult to compare. This is because almost every study has a different way of defining how many minutes or hours of wear time should count as a valid active day. By establishing standards for definitions of wear time, this could allow results across different patient populations to be compared more easily. This could also be solved by using a parameter other than step count as a measure of physical activity [41].

This review provides an overview of the frequently used wearables in oncology during therapy. Many of the wearables used have similar competencies, which might suggest the need to expand research into using more advanced wearables like smartwatches; according to Lu et al [2], very few clinical trials using smartwatches could be identified in 2016. Besides providing extended opportunities to measure variables, smartwatches also benefit from having a user-friendly display, which can make it feasible for patients to track their own activity status. Only 2 of the 25 reviewed studies used real-time feedback [24,40], and this feature might play an important role in motivating patients and possibly detecting worsening or severe symptoms earlier [11]. Technology allows plenty of different wearables to be applied in the oncology setting. However, it remains unknown if wearables can improve essential outcomes like overall survival or lead to other improvements in cancer treatment. Only future, well-designed research studies based on guidelines for this field with clinically relevant outcomes can help us decide when and where to apply these important tools.

This review provides an inventory for the status of wearables in clinical trials and can be used in addition to the CTTI studies database when designing new clinical trials with wearables [15].

### Conclusion

This review provides an overview of the use of wearable devices in oncology care for patients with solid tumors receiving antineoplastic treatment. We extracted data from studies monitoring patients with cancer and presented these results specifically regarding adherence, the device outcomes, and the types of wearables used.

We found that definitions of outcome measures and adherence varied across studies, and limited consensus among studies existed on which variables to monitor during treatment.

Less heterogeneity and better consensus in terms of use and establishing standards for definitions of wearable outcomes and adherence would improve the comparisons of outcomes among studies using wearables. Adherence and consistent definitions are crucial for drawing conclusions from data from wearable studies in oncology. Additionally, research using advanced wearable devices and active use of the data are encouraged to further explore the potential of wearables in oncology during treatment. Especially, randomized clinical studies are warranted to create consensus on when and how to implement in oncological practice.

## Appendix

Multimedia Appendix 1 Search strategy.

## Abbreviations

CTTI: Clinical Transformation Initiative
GI: gastrointestinal
GYN: gynecological
PA: physical activity
PRO: patient-reported outcome
RCT: randomized controlled trial
