# Supplementary material for: The Use of Wearables in Clinical Trials During Cancer Treatment: Systematic Review
Source: JMIR Mhealth Uhealth. 2020 Nov 11;8(11):e22006. doi: 10.2196/22006 (PMC7688381; doi:10.2196/22006)
Supplement: Multimedia Appendix 1 [file mhealth_v8i11e22006_app1.pdf]

PUBMED 03.10.2019

#1 "Neoplasms"[Mesh] OR "Medical Oncology"[Mesh] OR "Surgical Oncology"[Mesh]

#2 ("Wearable Electronic Devices"[Mesh]) NOT "Hearing Aids"[Mesh]

#3 #1 AND #2

#4 oncology OR cancer OR neoplasm

#5 "wearable device" OR "wearable" OR acceleromet* OR "actigraph" OR "activity monitoring”

#6 #4 AND #5

#7 #3 OR #6

Hits: 992

EMBASE 03.10.2019

1) exp neoplasm/

2) exp patient monitoring/

3) exp electronic device/

4) 2) OR 3)

5) 4) AND 1)

6) exp ambulatory monitoring/ OR exp telemedicine/

7) 5) AND 6)

8) exp *neoplasm/

9) exp *electronic device/

10) exp *patient monitoring/

11) exp *ambulatory monitoring/

12) exp *telemedicine/

13) 8) OR 9) OR 10) OR 11) OR 12)

14) 13) AND 7)

Hits: 289
